# Controversial role of herpesviruses in Alzheimer’s disease

**DOI:** 10.1371/journal.ppat.1008575

**Published:** 2020-06-18

**Authors:** Roberta Rizzo

**Affiliations:** Department of Chemical and Pharmaceutical Sciences, University of Ferrara, Ferrara, Italy; University of Michigan Medical School, UNITED STATES

## Introduction

The controversial hypothesis that microbes might trigger Alzheimer's disease (AD) has been debated for decades. Around 30 years ago, researchers in the United Kingdom discovered DNA of human herpes simplex virus 1 (HSV1) in postmortem brain samples of AD patients at much higher levels than in healthy brains, hinting that viral infection could be somehow involved in the disease [1]. Since then, researchers have bolstered the association between AD and HSV1 as well as other pathogens, particularly human herpesvirus 6 (HHV6A and HHV6B), yet proving causality has remained elusive.

Recent findings have shown that herpesvirus infections may induce amyloid beta (Aβ) production and deposition in the brain, resulting in antimicrobial activity [2]. Aβ oligomers might bind herpesvirus surface glycoproteins [3], possibly acting as a protective coating against neurotropic HSV1 and HHV6. Furthermore, the authors show that infection with herpesvirus seems to rapidly seed amyloid plaque deposition in a transgenic mouse model (5XFAD) and in a three-dimensional human neuronal cell-culture system [3]. These data lack confirmation by other groups.

## Herpesvirus and AD

Itzhaki and colleagues first identified brain infection with HSV1 as a risk factor for AD [1]. A high proportion of cognitively healthy elderly individuals and AD patients have HSV1 DNA in brain tissue after death [4]. HSV1-specific neutralizing ability of AD sera is reduced even in the presence of high quantity of Immunoglobulin G3 (IgG3). As IgG3 plays a key role in counteracting the ability of HSV1 to evade immune responses, these data reinforce the hypothesis of a pathogenetic role of HSV1 in AD [5]. The relevance of HSV1 brain infection for the development of AD is supported by studies on Apolipoprotein E (APOE)-ε4 transgenic mice, which show marked behavioral and pathological changes in HSV1-infected animals [6]. Repeated virus reactivations triggered progressive accumulation of AD molecular biomarkers in the neocortex and hippocampus and correlated with increasing cognitive deficits, becoming irreversible after seven cycles of reactivation [7]. HSV1 significantly accelerated amyloid formation of Aβ42 compared with noninfected cell supernatant and catalyzed the aggregation of the Aβ42, a major constituent of amyloid plaques in AD, in vitro and in animal models [8]. In vitro infection with HSV1 has been shown to affect processing and distribution of APP (amyloid precursor protein), the precursor to neurotoxic Aβ, by multiple mechanisms. Even the earliest event in HSV1 infection, binding of the virus to neuronal membranes, has been shown to enhance APP phosphorylation and Aβ accumulation [9]. While characteristic AD protein aggregation appears to indirectly result from mechanisms that facilitate viral entry and transport, HSV1 also directly inhibits autophagic processing through the actions of the viral protein ICP34.5 (infected cell protein 34.5) by binding Beclin-1, a critical protein in the initiation of autophagy [10]. Recently, a retrospective cohort study investigated the association between HSV infections and dementia and the effects of antiherpetic medications on the risk involved [11]. The authors enrolled a total of 33,448 subjects. This analysis revealed a hazard ratio of 2.564 for the development of dementia in the HSV-infected cohort relative to the non-HSV cohort. A risk reduction of dementia development in patients affected by HSV infections was found upon treatment with antiherpetic medications (HR: 0.092). The idea is being tested in a Phase II trial that is currently evaluating whether daily valacyclovir slows cognitive decline in 130 people with mild AD who tested positive for HSV1 or 2 (ClinicalTrials.gov Identifier: NCT03282916).

One recent study implicating herpesvirus in the pathogenesis of late-onset dementia has elicited much interest in the AD research. The study by Readhead and colleagues used a computational strategy determining that RNA levels of HHV6A and Human herpesvirus 7 (HHV7) and the DNA amount of HHV6A were increased in multiple brain regions of postmortem tissue samples from patients with AD compared with controls and that the increase correlated to amyloid plaque, neurofibrillary tangle densities, and clinical dementia ratings [12]. The relative risk of genes modulating the expression of regulators for Aβ precursor protein processing correlated to viral abundance. To note, these observations failed to detect the association with HSV1 infection, a risk factor for AD as showed by early work by Itzhaki and colleagues [1]. Similarly, the presence of Epstein–Barr virus (EBV) was not detected, in contrast with the results by Lin and colleagues, who analyzed DNA isolated from peripheral blood leukocytes and brain samples from AD patients [13]. DNA of HSV1, EBV, and HHV6, but not cytomegalovirus (CMV), was found. Interestingly, HHV6 was found in 70% of AD brains versus 40% of controls, while HSV1 was found at high levels in both. Carbone and colleagues found HHV6 in 23% of peripheral blood mononuclear cells compared to 4% of controls [14]. It is worth to notice that these papers [13, 14] employed only nested PCR to analyze the samples, without any confirmation using more reproducible methods with a lower risk of carryover contamination.

Jeong and Liu found some controversies in the results obtained by Readhead and colleagues [15]. In particular, they sustained that the quantitative methods employed by Readhead and colleagues were inappropriate for sparse datasets of the sort analyzed. They observed that the extremely low expression levels of viral RNA and DNA in the brain samples pose a problem of detection limits, suggesting that the published study does not prove a link between AD and viral load. Similarly, Agostini and colleagues failed to observe any relation between humoral immune response against HHV6 and AD and amnestic mild cognitive impairment (aMCI) [5]. Westman and colleagues found significantly lower HHV6 IgG reactivity in AD subjects compared to nondemented controls, whereas there were no differences in HSV, Varicella zoster virus (VZV), or CMV antibody levels between the groups [16]. Analysis of peripheral blood mononuclear cells presented comparable HHV-6 DNA levels in PBMCs of AD and nondemented subjects. Recently, Chorlton reported an alternative in silico analysis of Readhead and colleagues’ results [17]. The author showed that the modified ViromeScan used by Readhead and colleagues likely vastly overestimates viral read counts and in most cases (28 of 30 top viral readcount samples) identifies viral reads when probably none are present. Simulation showed this alternative method to be sensitive and the Readhead and colleagues method to be highly nonspecific.

Recently, Allnutt and colleagues used RNA sequencing data to search for viral transcripts [18]. For this, they made use of data that had been obtained from two different repositories: one consisting of 301 postmortem brain samples from the Mount Sinai Brain Bank and the second of 600 brain samples from the Religious Orders Study and Rush Memory and Aging Project. Both collections contained brains from both AD disease patients and healthy controls. Using the PathSeq algorithm, which is designed to plough through large amounts of human sequencing data and pick out microbial sequences, including 118 viruses, they revealed no statistically significant difference in the amount of HHV6A or HHV6B RNA between diseased and healthy brains in either of the two cohorts. For instance, in the Religious Orders group, HHV6A was detected in only one of 173 brains with confirmed AD and one of the 158 age-matched controls. Screens for other viruses, including EBV and several additional herpesviruses, also showed no significant difference between diseased brains or controls. The results were confirmed also testing 708 healthy or diseased brain samples for HHV6A or HHV6B viral DNA that had been extracted from. They found no significant difference between the AD samples and the controls.

## Potential mechanisms

**Fig 1:**
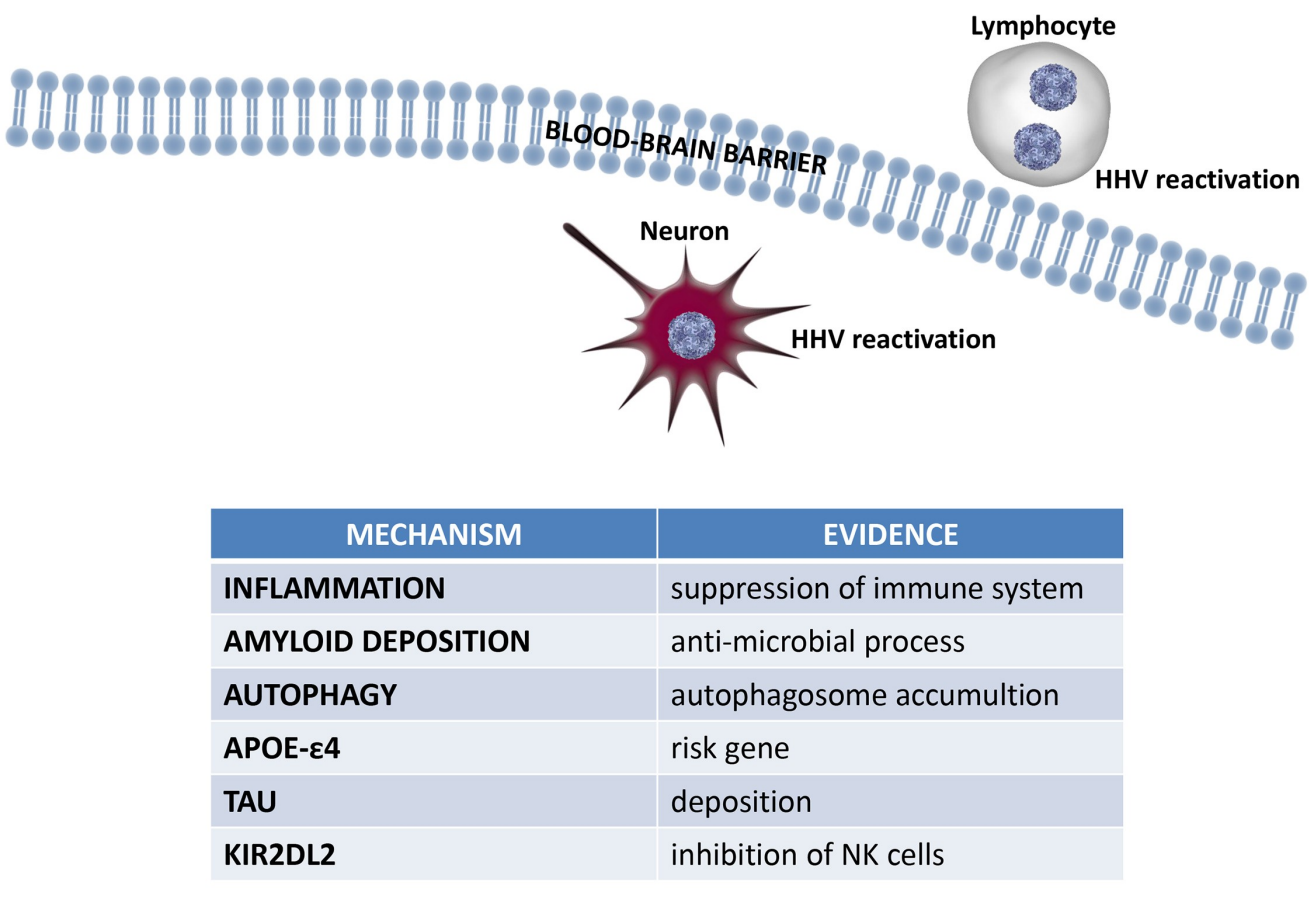
Potential mechanisms of HHV to increase the risk of AD. Both neuronal or peripheral blood lymphocyte HHV infection might be responsible of these modification via cell–cell contact or soluble factors. AD, Alzheimer's disease; APOE, Apolipoprotein E; HHV, human herpesvirus; KIR2DL2, killer immunoglobulin-like receptor 2.

Current evidence does not allow to establish a role for herpesviruses in AD etiopathogenesis. The controversial results point to a difference in research approaches and the need to clarify the possible role of roseola virus in the pathogenesis of the disease. Some of the results might be read with significant caution due to the presence of possible false positive correlations. Lacking an established association, at this moment we can only speculate on the mechanisms potentially used by herpesviruses in increasing the risk of AD. One possibility is that reactivation of herpesvirus in elderly individuals who are stressed or have suppressed immune system may damage the brain by increasing inflammation (**Fig 1**). Another possibility is the potential role of Aβ having antimicrobial properties. It is thought that Aβ may protect the brain by binding to and killing infectious microbes [2].

Interestingly, among the neurotropic herpesviruses, HSV1 and HHV6A have been reported to infect several cell types present in the central nervous system and dysregulate autophagy, a process required for homeostasis of cells, especially neurons [10]. Indeed, autophagosome accumulation, indicating an unbalance between autophagosome formation and degradation, has been observed in neurons of AD patients and may play a role in the intracellular and extracellular accumulation of Aβ and in altered protein tau metabolism. Moreover, herpesvirus infection of glial and microglial cells can increase the production of oxidant species through the alteration of mitochondrial dynamics and promote inflammation [10], another hallmark of AD. Recent results from my lab have shown that HHV-6A infection increases apolipoprotein E ε4, Aβ and tau expression in microglia cells and T cells [19, 20], suggesting a possible role in inducing expression of AD risk genes. Since HHV DNA is found in both AD and control brains, it is relevant to understand why an HHV infection might have different effects in different individuals: Some of them will develop AD, and others will not. In this context, we observed that KIR2DL2 gene increases the susceptibility to HHV infections in AD patients. KIR2DL2 is an inhibitory receptor expressed by NK cells [20, 21]. When it is silenced, NK cells regain the ability to recognize and kill HHV infected cells. The expression of this receptor might inhibit the activation of brain-infiltrating NK cells towards HHV infected neurons and might induce chronicization of neuronal infection, thus potentially contributing to the development of Aβ deposition and disease onset. Recently, a human brain-like tissue infected with HSV-1 showed the formation of amyloid plaque-like structures, supporting the role of viruses in AD [22] and proposing a model that will allow for future studies to identify potential downstream drug targets for treating AD. Uncovering this potential connection represents an interesting and exciting field of research. Certainly, unless new and more relevant animal models are developed and/or human clinical trials conducted, the association will remain speculative.
